# Effects of platelet‐rich plasma on the memory impairment, apoptosis, and hippocampal synaptic plasticity in a rat model of hepatic encephalopathy

**DOI:** 10.1002/brb3.2447

**Published:** 2021-12-02

**Authors:** Mahnaz Bayat, Azadeh Khalili, Gholamreza Bayat, Somayeh Akbari, Amirhossein Yousefi Nejad, Afshin Borhani Haghighi, Masoud Haghani

**Affiliations:** ^1^ Clinical Neurology Research Centre Shiraz University of Medical Sciences Shiraz Iran; ^2^ Department of Physiology‐Pharmacology‐Medical Physic School of Medicine Alborz University of Medical Sciences Karaj Iran; ^3^ Cardiovascular Research Center Alborz University of Medical Sciences Karaj Iran; ^4^ Department of Physiology The Medical School Shiraz University of Medical Sciences Shiraz Iran; ^5^ Faculty of Veterinary Medicine, Department of Veterinary Medicine Islamic Azad University of Kazeroon Shiraz Iran; ^6^ Histomorphometry and Stereology Research Centre Shiraz University of Medical Sciences Shiraz Iran

**Keywords:** field potential recording, hepatic encephalopathy, LTP, platelet‐rich plasma

## Abstract

**Objectives:**

In the present study, we aimed to determine whether intraperitoneal injection of platelet‐rich plasma (PRP) could have a neuroprotective effect on learning, memory, and synaptic plasticity impairment as well as hippocampal apoptosis in rats with hepatic encephalopathy induced by bile duct ligated (BDL).

**Methods:**

The rats were divided into four groups: the control, sham, BDL+ V (vehicle), and BDL+ PRP. The BDL rats were treated with PRP immediately after the surgery, and the injection was done every 3 days for 30 days. The passive avoidance and Morris water maze tests were used for the evaluation of learning and memory. The long‐term potentiation (LTP), basal‐synaptic transmission, and paired‐pulse ratio, as an index for measurement of neurotransmitter release probability, were evaluated by field‐potential recording. After taking a blood sample for assessment of the liver enzymes, the animals were sacrificed and their hippocampus was removed for evaluation of cleaved caspase‐3 by Western blot.

**Results:**

Serological assessment of the liver function showed that BDL severely impaired the liver function. Also, PRP treatment could partially improve the liver dysfunction along with recovery in fear memory and spatial learning memory performance, LTP, basal‐synaptic transmission, and neurotransmitter release probability. PRP‐treated rats also showed a significant reduction in neuronal apoptosis in the CA1 area.

**Conclusions:**

The results of this study suggest that PRP improves cognitive performance and synaptic plasticity in BDL rats via direct neuroprotective property and/or indirectly by improvement of hepatic dysfunction.

## Graphical abstract

According to our findings, the beneficial effects of platelet‐rich plasma in hepatic encephalopathy could be mediated via direct neuroprotective property via improvement of neurotransmitter release probability and basal‐synaptic transmission as well as indirect hepatohealing characteristics.

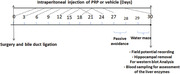

## 1 INTRODUCTION

Hepatic encephalopathy (HE) is a neuropsychiatric disorder seen in patients with acute or chronic liver diseases. A wide spectrum of neuropsychiatric disturbances following HE, ranging from minimal alteration in the personality to the complex changes in the brain function, leads to impairment in the intellect, cognition, motor activity, and coordination (Lemberg & Alejandra Fernández, 2009; Prakash & Mullen, 2010). The pathophysiological mechanism of HE is still not completely understood. Several clinical and experimental findings confirm the deleterious effects of hyperammonemia on neurological abnormalities induced by HE (Jiang et al., 2013; Kanamori et al., 1996; Quero et al., 1996). Some of the principal roles of hyperammonemia in the pathogenesis of HE have been reported in previous studies; they include inflammation (Rodrigo et al., 2010; Shawcross et al., 2004), disruption of the blood‐brain barrier integrity (Skowronska & Albrecht, 2012), an imbalance between the inhibitory and excitatory neurotransmission (Albrecht & Jones, 1999), and oxidative stress (Sinke et al., 2008). Such a massive disturbance in neuronal function strongly interferes with another physiological process such as development of normal long‐term memory and neuronal plasticity (Mohammadian et al., 2019; Rodrigo et al., 2010; Shabani et al., 2019). Despite the growing improvement in drug development research, no specific medicine has been introduced so far to prevent or cure HE. However, in the light of the known mechanisms involved in HE‐pathology, some targets can be considered as potential drug candidate in this situation. Administration of neurotrophic factors has shown therapeutic potentials for treating neurodegenerative abnormalities (Horita et al., 2006; Li & Stephenson, 2002; Schabitz et al., 2000; Weissmiller & Wu, 2012). The optimal concentrations of neurotrophic factors have strongly affected all of the normal neuronal functions such as proliferation, differentiation, neurogenesis, neuronal survival, hippocampal plasticity memory, and cognitive ability (Bekinschtein et al., 2011; Bergami et al., 2008; Scharfman et al., 2005). According to previous findings, platelets release several growth factors and cytokines (Jungbluth et al., 2014; Le Blanc et al., 2020; Pavlovic et al., 2016).

Platelet‐rich plasma (PRP) is one of the autologous products taken from whole blood that can easily be prepared. Following storage of the PRP in the freezer and subsequent thawing, a large number of platelets are lyzed, leading to the release of growth factors from the granules (Leiter & Walker, 2020). Platelet‐rich plasma treatment has shown numerous beneficial therapeutic effects in regenerative medicine, including cartilage repair (Kavadar et al., 2015), dermal healing (Marck et al., 2014; Ozcelik et al., 2016), enhancement of the recovery of peripheral nerve (Cho et al., 2010; Wu et al., 2016), and spinal cord injury (Chen et al., 2018). Moreover, the PRP therapy has been reported in the neuroinflammatory central nervous models such as vascular dementia (Bayat et al., 2020), multiple sclerosis (Borhani‐Haghighi & Mohamadi, 2019), Alzheimer's (Anitua et al., 2014), stroke (Hayon et al., 2013), and Parkinson's disease (Anitua et al., 2015); these models have diminished the inflammatory responses and promoted neuronal survival.

The above‐mentioned results recommend that PRP treatment, as a promising approach, may be useful in neurodegenerative disorders. The present study was designed to examine the neuroprotective effects of PRP in learning‐memory impairment, basal synaptic transmission, long‐term and short‐term synaptic plasticity, and hippocampal apoptosis following an experimental model of HE.

## 2 MATERIALS AND METHODS

### 2.1 Animals and grouping

Fifty adult male Sprague–Dawley rats (7–9 weeks old with body weight of 220–250 g) were used in this study. The animals were housed in optimum conditions such as free access to food and water, 12‐h light/dark cycle, and controlled temperature (23±1°C) and humidity (50 ± 10%). This study was conducted according to the procedures and guidelines approved by the Institutional Ethics Committee of Shiraz University of Medical Sciences. After 7 days of adaptation to the new environment, animals were divided randomly into 4 groups: the control (*n* = 8), sham (*n* = 10), Bile duct ligated rats which received vehicle (BDL + V [PBS, vehicle]; *n* = 15), and bile duct ligated rats treated with PRP (BDL+ PRP; *n* = 17). Eventually, 11 rats died on different days following BDL surgery, and the final grouping was as follows: the control (*n* = 8), sham (*n* = 9), BDL + V (*n* = 10), and BDL+ PRP (*n* = 12).

**FIGURE 1:**
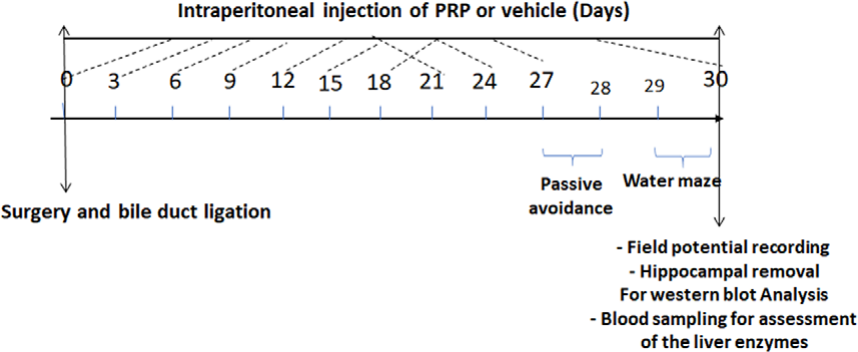
The schematic experimental protocol in the bile duct ligated rats (BDL) rats that received platelet‐rich plasma (PRP) or vehicle

In the BDL+ PRP group, the first dose of PRP was injected (intraperitoneal; i.p.) immediately after the surgery on day 0 and the injection continued on days 3, 6, 9, 12, 15, 18, 21, 24, 27, and 30. In all groups, the shuttle box was done on the 27th and 28th days, and the training sessions and probe trial of the Morris water maze were performed on the 29th and 30th days after the surgery. On the 30th day postsurgery, the field potential of the hippocampal CA1 region was recorded; then, at the end of the electrophysiology recording, 1 ml blood was taken for measurement of biochemical parameters, and the right hippocampus was dissected for western blot analysis (Figure 1).

### 2.2 The common bile duct ligation

The BDL model was used for induction of hepatic encephalopathy as mentioned in our previous study (Shabani et al., 2019). This model is recommended by the International Society for Hepatic Encephalopathy (Butterworth et al., 2009). At first, the animals were anesthetized by i.p. injection of ketamine (100 mg/kg) and xylazine (20 mg/kg); also, to expose the common bile duct, we performed mid‐line laparotomy. The common bile duct at two points was ligated with silk suture 5‐0, and between them was cut carefully. In the sham group, the surgical procedure was done with all of the surgical procedures without bile ducts obstruction. Finally, after closing the abdominal wall in two layers, the animals were allowed to recover from the anesthesia and then placed into separate cages until 24 h with free access to food and water. After 24 h, two or three rats were placed in each cage.

### 2.3 Preparation of platelet‐rich plasma

Twenty male Sprague–Dawley rats (250−300 g) were used for PRP extraction. By cardiac puncture, the whole blood of rats was obtained and mixed with 3.2% sodium citrate in special tubes. After 10 min centrifuge at 400× *g*, the supernatant in another tube centrifuge for an additional 10 min at 1000 × *g*. After discarding the upper layer, the remaining was collected as PRP and frozen at −80°C for use (Franco et al., 2012). By this method, compared with RBCs and leucocytes, the platelet percentage was over 90% in the PRP. To count the platelets, we used hemocytometers. Before counting, the diluted PRP was mixed with Rees and Ecker fluid at a ratio of 1:1, with a solution of 0.1 g of brilliant cresyl blue and 0.2 ml of 40% formaldehyde in 100 ml ultrapure water. In every 500 μl injection, there were 375,000,000 platelets (250 μl PRP + 250 μl PBS, i.p.) (Bayat et al., 2020). According to a previous study, intraperitoneal injection of PRP with the same dose showed beneficial therapeutic effects on cognition impairment following chronic cerebral hypoperfusion (Bayat et al., 2020). Finally, PRP was stored at −80°C maximum for 1 month before injection (McClain & McCarrel, 2019). The platelets in PRP were activated before injection, using the freezing and thawing method (İbrahim Eker et al., 2020).

### 2.4 Behavioral studies

#### 2.4.1 Passive avoidance test

For evaluation of the fear‐based conditioned avoidance learning and memory, a shuttle box was used according to our previous published studies (Bayat et al., 2020; Mohammadian et al., 2019). The shuttle box has two bright and dark halves divided by a guillotine door. On the 27th day after surgery, the learning trials were performed. At first, each rat was placed on the bright side upon entrance to the dark side; a 50 Hz electrical foot‐shock (2 s, 0.5 mA) was delivered for the development of fear conditioning. This stage was repeated every 5 min until the rats did not pass in the dark halve. The number of electrical shocks that were given to each animal was considered as a learning index. The step‐through latency (STL) is the time the animals stay in the bright halve before crossing through the gate from the bright to dark halves. On the 28th day postsurgery, the STL time was recorded as fear memory index without any punishing electric shock.

#### 2.4.2 Morris water maze

For assessment of spatial learning and memory, Morris water maze test was used in the same way as our previously published study (Shabani et al., 2019). The water maze tank consisted of a circular pool filled with water (140 cm wide and 45 cm high), the water temperature was 21−23°C. The animals were blindly tested by an experimenter, and data were collected by a video image motion analyzer (Ethovision, Noldus Information Technology; the Netherlands). On the 29th day postsurgery, one training session was performed while a platform was placed 1.5 cm above the water surface of a circular pool for evaluation of visible memory. Subsequently, during the three blocks, each of which consisted of four trials, the acquisition of learning was evaluated while the platform was submerged in the target quadrant. In this stage, the time and distance spent to find the hidden platform were considered as the indexes for spatial learning. The maximum duration of each trial, intertrial interval, and interblock interval were considered 60 s, 35 s, and 30 min, respectively. On the 30th day postsurgery, the spatial memory was evaluated in the probe trial. In this trial, the rat was allowed to swim for 60 s while the platform was removed. A higher percentage of the time and distance spent in the target quadrant showed better spatial memory retention.

### 2.5 Field potential recording

On the 30th day postsurgery, after the probe trial, the field potential of the hippocampal CA1 region was recorded for each rat, as described previously in more detail (Bayat et al., 2020; Firouzjaei et al., 2019). Briefly, after intraperitoneal injection of 1.5 g/kg urethane and fixation of anesthetized rats in a stereotaxic apparatus, two small holes were drilled in the skull at the Schaffer collateral pathway (−4 AP and 3 L) and the CA1 region (−3 AP and 2 L); then, 0.2 mm diameter electrodes (Advent, UK) were carefully inserted. The recorded field excitatory postsynaptic potential (fEPSP) from CA1 had a typical waveform shape with amplitude and slope. This shape of fEPSP is considered as the confirmation of the correct position of the electrode in the CA1. After 25‐min rest, the input/output curve was plotted by incrementing the stimulation intensity from 50 to 1200 μA. For estimation of the intensity for baseline recording and high‐frequency stimulation (HFS), 40% and 80% of the maximum amplitude responses were calculated from the input–output curve in each rat, respectively. Before LTP induction for 25 min, the amplitude of the baseline fEPSP was recorded. Also, the short‐term plasticity was evaluated by calculation of paired‐pulse ration (pulse 2/pulse 1) at interstimulus intervals of 25, 50, 100, 150, 200, and 250 ms before and after the delivery of HFS. The HFS was composed of 3 trains at 0.1 Hz in each container with 20 pulses at a frequency of 200 Hz. Following induction of HFS, the stimulus intensity was decreased to 40% of the maximum response, and recording was performed for 60 min. The level of LTP induction was evaluated by calculating the percentage change of fEPSP amplitude after HFS to the baseline value.

### 2.6 Western blot analysis

We used Western blot analysis of cleaved caspase‐3 protein, as an index of apoptosis (Firouzjaei et al., 2019). At the end of the experiment, the dissected right hippocampus was rapidly placed in a liquid nitrogen tank, and then stored at −80°C. Following Bradford assay, for electrophoresis, the samples were placed into the gel and transferred onto a PVDF membrane. The PVDF was incubated overnight with anticleaved caspase‐3 (1:1000 SC‐7148, Sant Cruz) or anti‐β‐actin (1:1000, ab8227, Abcam); then, the membranes were incubated with the Goat antirabbit horseradish peroxidase‐conjugated (1:10,000; ab6721), as the secondary antibody (Abcam), for 1 h at room temperature. Through a gel documentation system, the protein level (densitometry) was quantified.

### 2.7 Measurement of biochemical parameters

At the end of the recording (before decapitation), 1 ml blood was taken for measurement of biochemical parameters. Albumin (ALB), alanine aminotransferase (ALT), aspartate aminotransferase (AST), alkaline phosphatase (ALP), and total bilirubin (TBIL) were measured using standard methods.

### 2.8 Statistical analysis

Kolmogorov–Smirnov test was used to test the normal distribution. One‐way ANOVA with Tukey's posthoc test was used to compare the means of 4 groups with normal distribution; then, in the case of nonnormally distributed data, the Kruskal–Wallis test with Dunn's multiple comparisons test was applied for comparison. All values are expressed as Mean ± SEM. Using paired *t*‐test, we compared the normalized amplitude of EPSP before and after HFS and the levels of paired‐pulse ratio changes before and after HFS delivery. We compared the traveled distance and time in 3 blocks of the Morris water maze and fEPSP changes after delivery of high‐frequency stimulation using two‐way repeated‐measures ANOVA test to evaluate the time and interaction (time and group) effects. PRISM.6 software was used for analyzing all the data, and the level of significance was set at *p *< .05.

## 3 RESULTS

Overall, statistical analysis showed that there was no significant difference between the control and sham groups. Thus, we reported only comparisons with the sham group.

### 3.1 PRP improved the passive avoidance memory task in bile duct ligation rats

**FIGURE 2:**
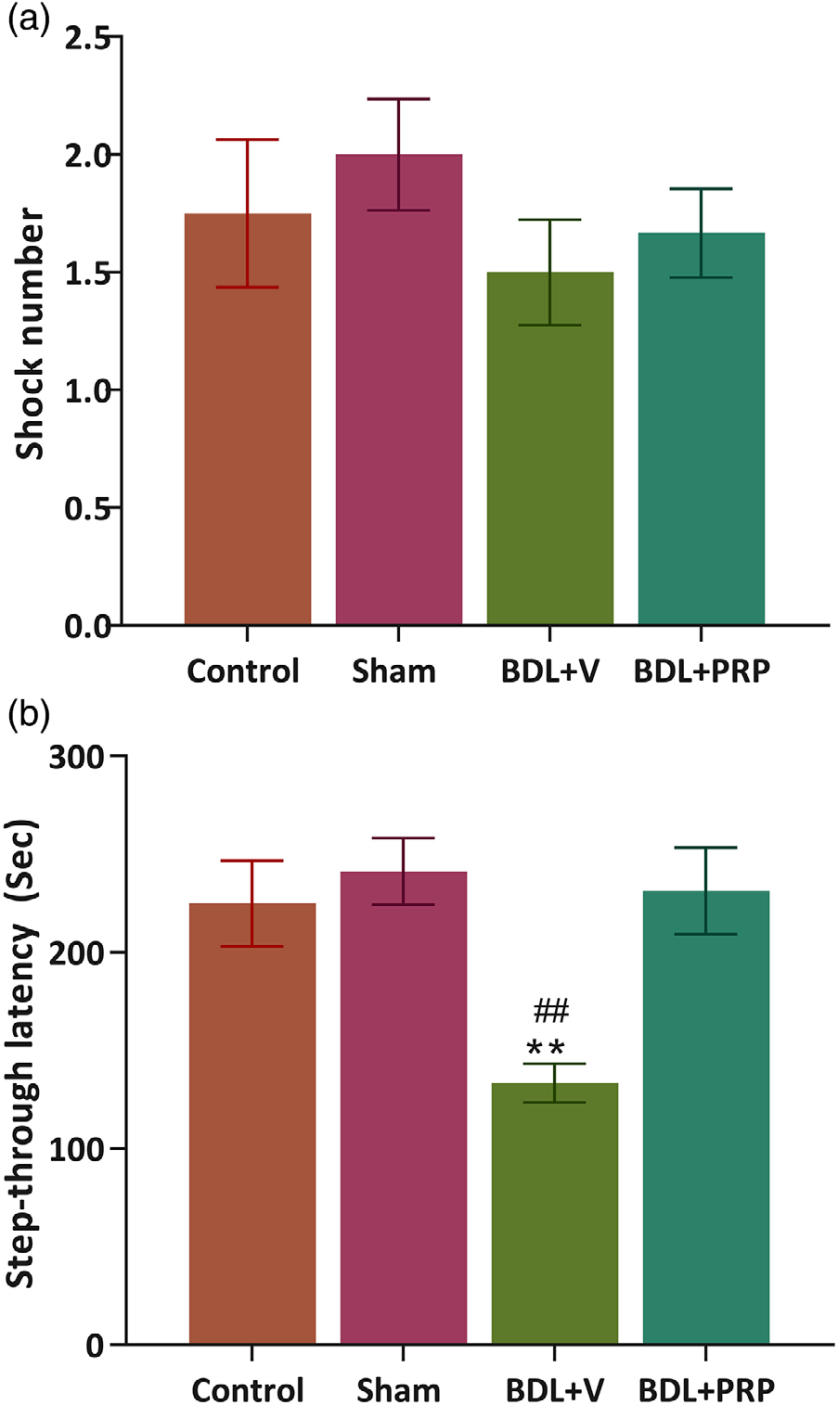
Effects of platelet‐rich plasma (PRP) on passive avoidance memory. All groups received the same number of shocks (a). Treatment with PRP resulted in a significantly higher STL time when compared to STL values seen in the BDL + V group (b). The values are shown as mean ± SEM. Significant differences with respect to the sham (**p < .01) and BDL+ PRP (## *p *< .01). Control (*n* = 8), sham (*n* = 9), BDL + V (*n* = 10), and BDL+ PRP (*n* = 12), using one‐way ANOVA with post hoc test

The results showed no significant differences between the shocking number applied in all groups (*F*(3, 35) = 0.76; *p* = .51). Thus, passive avoidance learning seemed to be similar in all groups (Figure 2a). In the BDL + V rats, the STL time significantly decreased (133.5 ± 9.8 s; *p* < .01) as compared to this value in the sham group (241.4 ± 16.9 s). Further, treatment with the PRP resulted in greater STL time in the BDL rats (231.3 ± 21.9 s) than that seen in the BDL + V group (*p* < .01) (Figure 2b), suggesting an improvement in fear‐memory performance (*F*(3, 35) = 7.25; *p* = .0007).

### 3.2 PRP improved the spatial learning and memory task in the bile duct ligated rats

**FIGURE 3:**
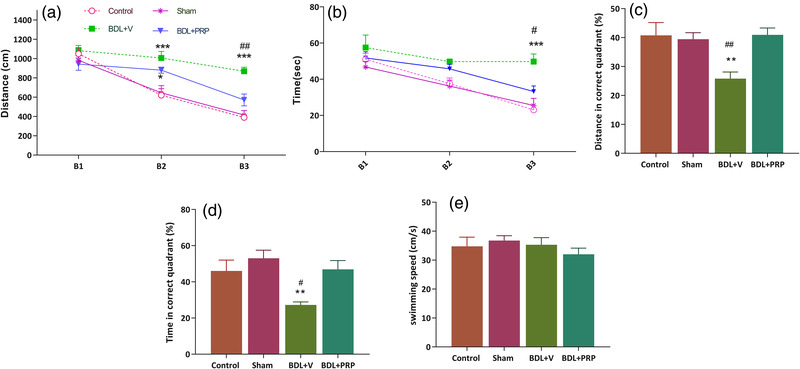
Effects of platelet‐rich plasma (PRP) on spatial learning and memory in the Morris water maze. PRP recovered the spatial learning performance of the bile duct ligation (BDL) rats and decreased the traveled distance (a) and time (b) to the level of normal animals during the third block (two‐way ANOVA). PRP therapy was associated with an increase in the percentage of the traveled distance (c) and time (d) spent in the probe trial after removal of the platform in the correct quadrant. (one‐way ANOVA with post hoc test). Swimming speed (e) in all groups was the same. Significant differences with respect to the sham (**p < .01, ***p < .001) and BDL + PRP (#*p* < .05, ##*p* < .01). The values are shown as mean ± SEM. Control (*n* = 8), sham (*n* = 9), BDL + V (*n* = 10), and BDL+ PRP (*n* = 12)

During the training phase of the Morris water maze task, the progression of spatial learning was evaluated across 12 trials conducted in three blocks by the distance and time spent to find the hidden platform by each rat (Figure 3a–b). During the second and third blocks, the BDL + V rats swam a significantly (*p* < .001) longer distance (1006.2 ± 68.7 cm) and (869.5 ± 40.6 cm), respectively, to reach the platform, compared to the sham group (Figure 3a). Besides, during the third block, the BDL + V rats required significantly more time (49.750 ± 4.2 s; *p* < .001) compared to the sham group (25.5 ± 3.9 s) to reach the platform. However, during the third block in the BDL+PRP group, similar to the normal rats, we found a progressive decrease in the distance (571.5 ± 62.1 cm) and time (33.2 ± 3.03 s) needed to find the platform compared to the BDL + V group (*p* < .01, *p* < .05, respectively; two‐way ANOVA, *F*(3, 12) = 8.218; *p* = .0031).

In the probe trial, the percentage of swimming distance and time decreased in the correct quadrant in the BDL + V group (25.8 ± 2.2%, and 27.2 ± 1.7%, respectively) compared to the sham (39.4 ± 2.2% and 53 ± 4.5%, respectively; *p* < .01) group (Figure 3c and d). In the BDL+ PRP group, the higher swimming distance (40.9.1 ± 2.4%) and time percentage (46.9 ± 4.8; *p *< .01, *p *< .05, respectively) in the correct quadrant relative to the BDL + V group showed that PRP therapy improved the memory in BDL rats. Additionally, all groups showed the same swimming speed (Figure 3e).

### 3.3 Field potential recording

#### 3.3.1 PRP improved the basal synaptic transmission of the CA1 neurons in the bile duct ligated rats

**FIGURE 4:**
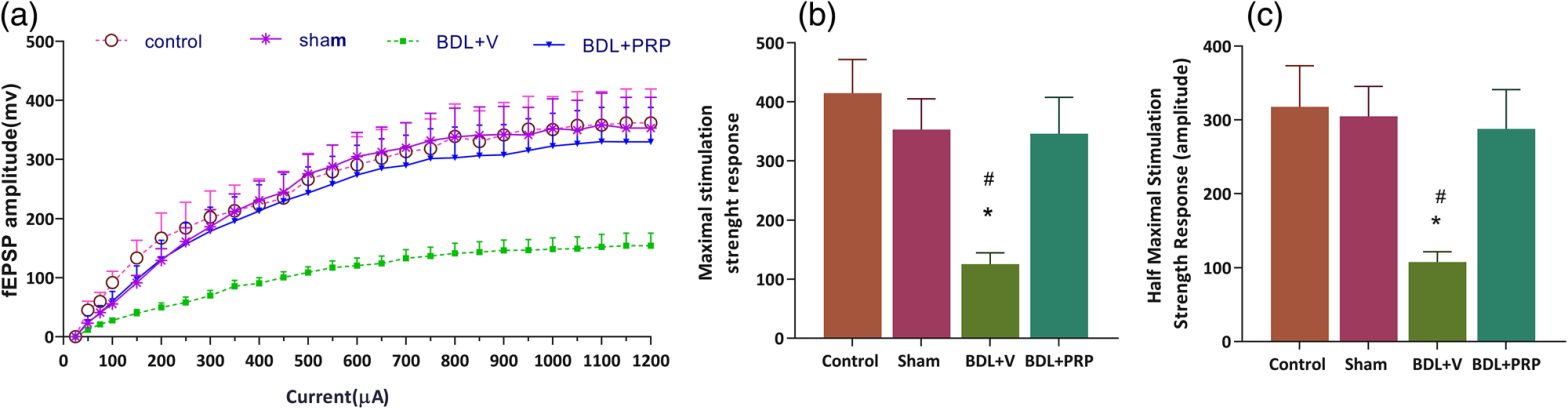
The basal synaptic transmission of the hippocampal CA1 neurons was recovered with platelet‐rich plasma (PRP) therapy in bile duct ligation (BDL) rats. The input/output curve (a), the maximum stimulation strength response amplitudes (b), and the half‐maximum stimulation strength response amplitudes (c) are expressed as means ± SEM. Significant differences for the sham (*p < .05, **p < .01) and BDL+ PRP (#*p* < .05). Control (*n* = 8), sham (*n* = 8), BDL + V (*n* = 9), and BDL+ PRP (*n* = 10)

For evaluation of the effects of bile duct ligation and PRP therapy on the baseline synaptic transmission in the first set of field potential recordings, the input–output curve was designed by recording fEPSP responses in the CA1 region to progressive increases in the stimulus intensity (50−1200 μA). Figure 4a shows that there was a noticeable right shift in the slope of I/O curves in the BDL + V group compared to those of the sham group; also, PRP administration exhibited a significant left shift in the I/O curve. Therefore, for more detailed comparisons for each group, the maximal and half‐maximal fEPSP slope was calculated from the I/O curve. Further analysis showed that the max (125.7 ± 19.2) and half max (107.6 ± 13.8) stimulation strength responses in the BDL + V were significantly (*p* < .05) decreased compared to the sham group. It was found that in the BDL+PRP group, the max (346.9 ± 61.1) and half‐max (287.4 ± 53.4) responses were significantly greater compared to those in the BDL+ V (*p* < .05) *F*(3, 31) = 6.161; *p* = .0021 and *F*(3, 31) = 4.9; *p* = .0065, respectively (Figure 4b and c).

#### 3.3.2 The effects of PRP administration on short‐term plasticity in bile duct ligated rats

**FIGURE 5:**
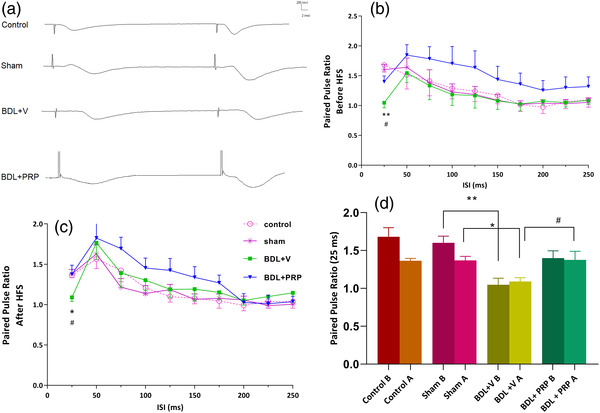
The effects of platelet‐rich plasma (PRP) administration on the short‐term synaptic plasticity in bile duct ligation (BDL) rats. The linear graph of PPR plotted for different ISIs (25−250 ms) before (a) and after (b) HFS delivery. The values are shown as mean ± SEM. Therefore, there was significant depression in before and after HFS paired‐pulse ratio (ISI 25 ms) in the BDL + V compared to the sham group. PRP treatment could significantly recover the paired pulse ratio. Nonsignificant depression in comparison of before with after HFS ratio for ISI of 25 in the sham and control groups (paired *t*‐test) and one‐way ANOVA comparison between different groups in before HFS ratio or after HFS ratio. Control (*n* = 8), sham (*n* = 8), BDL + V (*n* = 9), and BDL+ PRP (*n* = 10). Significant differences for the sham (*p < .05, **p < .01) and BDL+ PRP (#*p* < .05)

Short‐term synaptic plasticity was evaluated by the paired‐pulse ratio pre‐ and post‐HFS (Haghani et al., 2016). The sample traces of the paired‐pulse ratio at ISI of 25 ms are shown in Figure 5a. Also as shown in Figure 5b and c, the bile duct ligated rats had significantly lower values of paired‐pulse ratio at ISI 25 ms before HFS and after HSF with respect to those of the sham group (1.06 ± 0.07 vs. 1.5 ± 0.07, *p* < .01) (1.09 ± 0.05 vs. 1.37 ± 0.05, *p* < .05), respectively. Treatment with PRP significantly increased pre‐HFS paired‐pulse ratio (ISI 25 ms) compared to BDL + V (1.4 ± 0.09 vs. 1.06 ± 0.07, *p* < .05) *F*(3, 31) = 8.4; *p* = .0003 and post‐HFS paired‐pulse ratio (ISI 25 ms) compared to BDL + V (1.37 ± 0.11 vs. 1.09 ± 0.05, *p* < .05) *F*(3, 31) = 4.5; *p* = .0096 (Figure 5b and c).

Moreover, in the sham group, the within group paired *t*‐test analysis showed that delivery of HFS resulted in nonsignificant suppression in paired‐pulse ratio values at post‐HSF compared to pre‐HSF at ISI 25 ms (Figure 5d). However, the BDL prevents this suppressive effect of HFS on paired‐pulse ratio, and PRP could not recover this normal suppression effect in short‐term plasticity (Figure 5d).

#### 3.3.3 Long‐term synaptic plasticity improvement by PRP administration at Schaffer collateral–CA1 synapse in bile duct ligated rats

**FIGURE 6:**
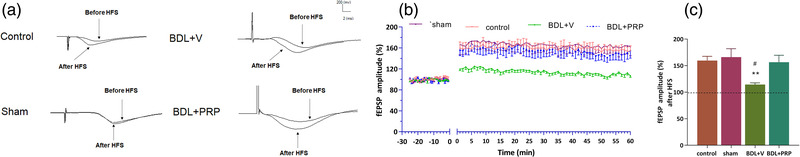
The positive effects of platelet‐rich plasma (PRP) therapy on the long‐term synaptic plasticity (LTP) in the bile duct ligation (BDL) rats. The level of LTP induction in sample traces of responses (a). The percent change of fEPSP amplitude compared to the baseline after HFS stimulation (b). The means of the percentage change in fEPSP amplitude after HFS in each group (c). Significant differences with respect to the sham (**p < .01) and BDL+PRP (#*p* < .05). The values are shown as mean ± SEM. Control (*n* = 8), sham (*n* = 8), BDL + V (*n* = 9), and BDL+ PRP (*n* = 10)

The results showed that the ability of synapse for LTP potentiation was impaired after bile duct ligation. Thus, the mean of fEPSP amplitudes after HFS in the BDL + V group (113.5 ± 3.5%) was significantly (*p* < .01) lower than the value of the sham group (171.2 ± 17%). However, a significant degree of LTP recovery was recorded in the BDL + PRP group (154.1 ± 11.9) compared to the BDL + V group (*p* < .05) *F*(3, 31) = 5.0; *p* = .0056 (Figure 6a–c). Furthermore, the size of LTP in the BDL + PRP and sham groups was found to have no significant difference (Figure 6c).

### 3.4 The effects of treatment with PRP on liver dysfunction induced by bile duct ligation

Table 1 shows that bile duct ligation could severely impair liver function with much higher levels of aspartate aminotransferase (AST), alanine aminotransferase (ALT), total bilirubin (T.BIL) (*p* < .001), and alkaline phosphatase (ALP) (*p* < .01), as well as significantly lower level of albumin (*p* < .001) compared to the sham group. Also, treatment with PRP could partially recover the liver function with decreased levels of aspartate aminotransferase (AST), and ALT (*p* < .05) and a significant increase in serum level of ALB compared to to BDL + V (*p* < .01). PRP administration could not improve the ALP and T.BIL.

**TABLE 1:** The effect of bile duct ligation (BDL) and platelet‐rich plasma (PRP) treatment on biochemical parameters

Biochemical parameters					
Groups	ALB (g/dl)	AST (U/L)	ALT (U/L)	ALP (U/L)	T.BIL (micromole/L)
Control	2.288 ± 0.11	123.4 ± 31.15	49.38 ± 3.00	632.6 ± 80.78	0.22 ± 0.0
Sham	2.233 ± 0.16	115.3 ± 19.02	51.11 ± 4.05	570.1 ± 63.41	0.22 ± 0.01
BDL+ V	1.140 ± 0.12***	1925 ± 416.8***	189.3 ± 14.25***	1076 ± 65.12**	6.98 ± 1.10***
BDL+ PRP	1.81 ± 0.09^##^	1068 ± 145.9^#^	130.8 ± 20^#^	813 ± 90.2	5.31 ± 1.16

*Note*: The values are shown as mean ± SEM. Significant differences respect to the sham (**p < .01 and ***p < .001) and relative to the BDL + PRP (#*p* < .05, ##*p* < .01) groups. Control (*n* = 8), sham (*n* = 9), BDL + V (*n* = 10), and BDL+ PRP (*n* = 12).

### 3.5 The effects of treatment with PRP on apoptosis induced by bile duct ligation

**FIGURE 7:**
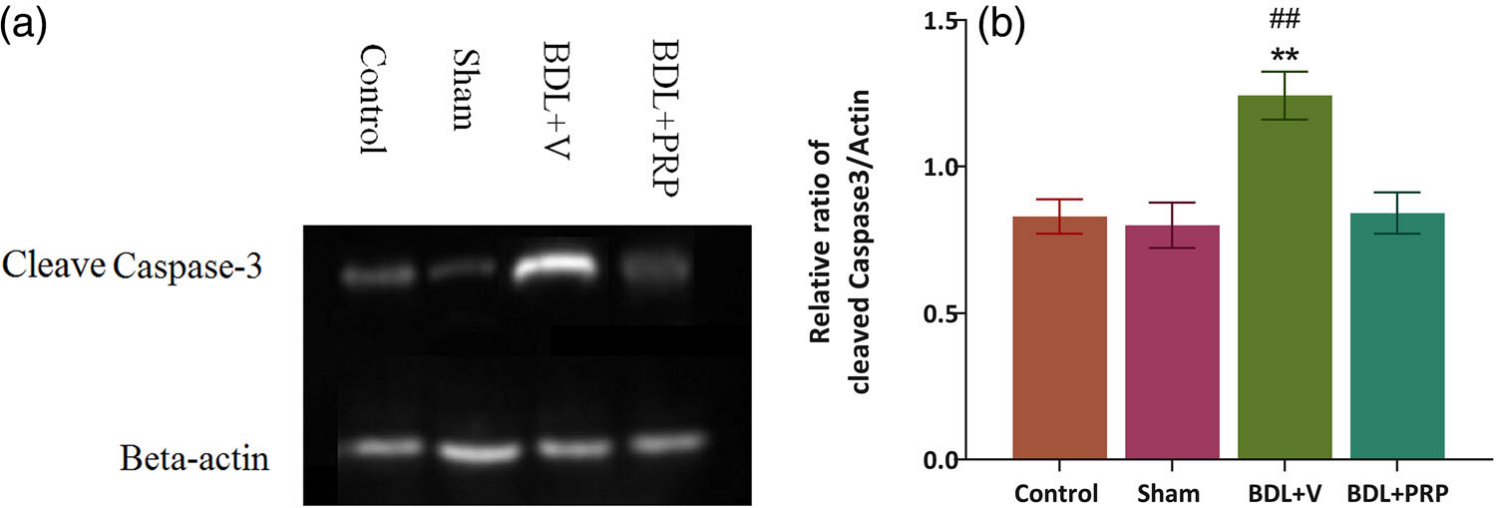
Bile duct ligation (BDL) increased cell apoptosis, and the platelet‐rich plasma (PRP) reduced apoptosis in the hippocampus. Western blot image (a) and graph of cleaved caspase‐3/β‐actin for different groups (b). Significant differences relative to the sham (**p < .01) and BDL + PRP (##*p *< .01) groups. The values are shown as mean ± SEM. Control (*n* = 5), sham (*n* = 5), BDL + V (*n* = 5), and BDL+ PRP (*n* = 7)

BDL induced apoptosis and the relative ratio of cleaved caspase‐3/β‐actin significantly increased (Figure 7a and b) in the BDL + V group compared to the sham group (1.24 ± 0.08 vs. 0.79 ± 0.07, *p* < .01). Besides, PRP administration significantly decreased the ratios of cleaved caspase‐3/β‐actin in the BDL + PRP (0.84 ± 0.07) compared to the BDL + V group (*p* < .01) *F*(3, 18) = 7.63; *p* = .0017.

## 4 DISCUSSION

The main objective of the present study was to examine the therapeutic efficacy of intraperitoneal injection of platelet‐rich plasma on cognitive and synaptic plasticity impairment in cirrhosis‐induced hepatic encephalopathy (HE). Treatment with PRP in this model was associated with remarkable improvement in passive avoidance and spatial learning and memory tasks. Moreover, field potential data analysis also exhibited an improvement in both basal synaptic transmission, and short‐ and long‐term synaptic plasticity of the CA1 neurons. Serological assessment of the liver function also showed the partial protective effects of PRP on liver performance which was characterized by reduced aspartate aminotransferase (AST) and alanine aminotransferase (ALT) along with an increase in the plasma albumin level. Thus, in our study cognition improvement along with recovery in hippocampal synaptic plasticity in the bile duct ligated rats following long‐term PRP treatment might be attributed to the primary neuroprotective effect of PRP on the brain and/or secondary effect of PRP following liver functional enhancement. The most important evidence to support the primary neuroprotective effect of PRP and different growth factors on the brain will be mentioned in the following section. According to previous studies, the peripheral and central routes of PRP administration could show neuroprotective effects in neurodegenerative models (Bayat et al., 2020; Borhani‐Haghighi & Mohamadi, 2019; Hayon et al., 2013).

Also, previous studies demonstrated a neuroprotective effect of different growth factors such as brain‐derived neurotrophic factor (BDNF) (Schabitz et al., 2000; Zhang & Pardridge, 2006), Fibroblast growth factors (FGF) (Fisher et al., 1995; Li & Stephenson, 2002), and Glial cell line‐derived neurotrophic factor (GDNF) (Horita et al., 2006) when delivered intravenously in experimental models. On the other hand, BBB breakdown which has the potential to facilitate the blood‐brain permeability (Wang et al., 2013) is a serious concern in HE patients (Cui et al., 2013) and the BDL model following the vasogenic brain edema (Chen et al., 2012; Dhanda & Sandhir, 2018). There is also evidence in support of the primary effect of PRP on the brain, explainingdifferent methods for transport of the growth factors across the BBB. Nerve growth factor (NGF) can cross the blood‐brain barrier by receptor‐mediated transport (Friden, 1994). The basic FGF (FGF2) is transported from blood to the brain by adsorptive transcytosis (Deguchi et al., 2000) whereas FGF21 by simple diffusion crosses the BBB (Hsuchou et al., 2007). Insulin‐like growth factors (IGF)‐1 and IGF‐2 can transport from the blood into the brain at the BBB via receptor‐mediated transcytosis mechanisms (Duffy et al., 1988). The intact BDNF in the peripheral circulation by a high‐saturable and high‐capacity transport system can cross the BBB (Pan et al., 1998). Jiang et al. (2014) reported enhancement of the blood‐brain barrier permeability after intravenous injection of vascular endothelial growth factors (VEGF) in mice. VEGF stimulates endocytosis and transcytosis from BBB and can facilitate the transfer across the BBB (Nakayama & Berger, 2013).

In this work, the BDL caused fear memory deficit and spatial memory impairment as evidenced by the decrease in the STL time in the passive avoidance test and decrease in the percentage of time and distance in the probe trial of Morris water maze, which supported the previous animal studies (Huang et al., 2009; Mohammadian et al., 2019). The hippocampus plays a main role in modulating the spatial learning and memory in the Morris water‐maze task, and for contextual learning, the animal needs more brain regions from the hippocampus to the parahippocampal area (Burwell et al., 2004).

As previously disused, hyperammonemia and accumulation of toxic products are some of the main underlying causes of HE (Ciećko‐Michalska et al., 2012; Jayakumar & Norenberg, 2018). Hyperammonemia induced neuroinflammation (Norenberg et al., 2007) and increased mitochondrial permeability in astrocytes (Alvarez et al., 2011) and, as a consequence, hyperammonemia and inflammation‐induced mitochondrial dysfunction play key roles in the development of cognitive impairment. In different brain areas, susceptibility to hyperammonemia varies significantly. Heavy activation of microglia (resident brain innate immune cells) following hyperammonemia in the cerebellum and the mild microglial activation in the hippocampus, piriform cortex, and corpus callosum have been reported in BDL rats (Rodrigo et al., 2010). Microglial activation is associated with impairment in spatial learning memory (Wadhwa et al., 2017; Wang et al., 2015), and it plays an active role in modifying neuronal plasticity (Innes et al., 2019; Kim et al., 2013). Microglia through apoptosis can shape the adult hippocampal neurogenesis (Sierra et al., 2010). Following the evaluation of cellular response in in‐vitro models, PRP had effects in terms of decreasing inflammation in the microglia, reducing apoptosis in the neural progenitor cells, and stabilizing the neuronal synapses (Delgado et al., 2021).

In the next series of experiments, we used field potential recording and Western blot analysis to find neuroprotective mechanisms of PRP on synaptic plasticity, synaptic transmission, and rate of apoptosis of the CA1 neurons. We found a downward and rightward shift of the I/O curve in the BDL + V group, which represents a reduction in basal synaptic transmission (BST) of the CA1 neurons. This result is consistent with those of previous studies, indicating that the BDL strongly depressed BST of the hippocampal and Purkinje neurons (Aghaei et al., 2016; Mohammadian et al., 2019; Tahamtan et al., 2017).

There is a feedback loop between the microglia and neurons; microglia activation was mediated by the chemokine release from the neurons, and the chemokine led to synaptic depression in the neurons (Wu et al., 2015). Moreover, evaluation of intrinsic electrophysiological properties of the hippocampal neurons in the BDL rats has shown that depression in BST is caused by increases in IA, KCa2+, and Ca2+ currents (Shabani et al., 2019; Tahamtan et al., 2017). In the presence of chronic hyperammonemia, the action of GAT3 (GABA transporters) on the surface of the astrocytes and microglial cells can be reversed, leading to an increase in GABAergic tone in the synaptic cleft (Hernandez‐Rabaza et al., 2016). A maintained balance between excitatory and inhibitory synaptic transmission is needed for the function of neuronal networks in the CNS. Inhibition is mediated mainly by the neurotransmitter γ‐aminobutyric acid (GABA) (Levinson & El‐Husseini, 2005). Thus, it is likely that an increase in the GABAergic tone in the BDL may also cause depression in synaptic transmission, which is represented by a downward shift of the I/O curve in field potential recording. In our study, administration of PRP has been associated with the recovery of hippocampal BST; this result is consistent with that of a previous study that reported the beneficial effect of PRP on BST depression in the vascular dementia model (Bayat et al., 2020). Zafra et al. (1991) reported that BDNF expression was suppressed with activation of GABAergic transmission in the rat hippocampus. In addition, treatment with recombinant BDNF improves the depressed basal synaptic transmission and LTP in a hippocampal slice of BDNF knockout mice at the Schaffer collateral–CA1 synapse (Patterson et al., 1996). On the other hand, large quantities of BDNF can release following platelets activation into circulation (Le Blanc et al., 2020). It can be suggested that improvement in basal synaptic transmission is probably mediated by the links between PRP and BDNF or/and intrinsic electrophysiological properties of the CA1 neurons or/and microglial cells. However, we found a significant depression at the paired‐pulse ratio with interspike interval (ISI) 25 ms before and after HSF in the BDL + V group compared with the normal rats. This finding is consistent with that of a previous study (Mohammadian et al., 2019). The paired‐pulse ratio is a good index for evaluation of presynaptic release probability (Dobrunz & Stevens, 1997; Oleskevich et al., 2000). PRP therapy led to significant increases in a pre‐ and post‐HSF paired‐pulse ratio compared to the BDL + V group. It is well‐known the level of paired‐pulse ratio tends to reduce in synapses with the high initial release probability (Foster et al., 2002). Enhancement in the number of available vesicles and release probability of neurotransmitters was reported following peripheral inflammation (Toyoda et al., 2009). Thus, it is probable that inflammation which is a constant event in BDL may play an important role in the pathogenesis of high initial release probability. Previous studies demonstrated the anti‐inflammatory effects of the platelet and growth factors (Circolo et al., 1990; Merly et al., 1998; Rios et al., 2015). Therefore, probably the enhanced paired‐pulse ratio by PRP therapy that reduced the initial release probability has resulted from its anti‐inflammatory effects.

Finally, we recorded LTP as well‐known synaptic plasticity in the cellular mechanism of learning and memory in the hippocampus. We found impairment in LTP induction in the bile‐duct ligated rats along with a behavioral disturbance in passive avoidance and MWM tasks. Previous studies have demonstrated the LTP impairment of the BDL model (Mohammadian et al., 2019). Moreover, impairment of glutamate–nitric oxide–cGMP pathway induced by HE is considered as a confounding factor that interferes with the normal neuronal plasticity process. Therefore, short‐ and long‐term potentiation which is formed by this process is distorted (Monfort et al., 2001). Interestingly, our data indicated that the administration of PRP could attenuate the suppressive effect of BDL on LTP. Administration of PRP results in the release of neuronal signaling molecules and growth factors, which serve as a neuroprotective agent; according to previous studies, PRP regulates the neuronal differentiation and survival (Alcaraz et al., 2015; Jesús, 2017) with modulating effect in LTP recovery (Caraci et al., 2015; De Rossi et al., 2016; Dyer et al., 2016; Kelly et al., 1998; Lu et al., 2008).

The positive effects of neurotrophic factors on the induction of neuronal plasticity and improvement of cognitive and memory deficit have been strongly demonstrated (Miranda et al., 2019). On the other hand, platelets via the modulation of endogenous neurogenesis could have important implications in maintaining the brain plasticity to recover cognitive function in neuropsychiatric disorder and aging (Leiter & Walker, 2019). Anti‐inflammatory properties along with the presence of several trophic factors play an important role in the therapeutic effects of PRP in neurological disorders (Jesús, 2017; Sánchez et al., 2016). Moreover, the assessment of the cleaved caspase‐3/β‐actin relative ratio in this study indicated a significant antiapoptotic activity of PRP characterized by a marked reduction of the relative ratio. Similar findings regarding the antiapoptotic effects of PRP were previously reported by others (Fukaya et al., 2012; Moussa et al., 2017). Thus, administration of the rich source of trophic factors in memory‐impaired cirrhotic animals could be mediated directly via central anti‐inflammatory and antiapoptotic effects. Our findings, consistent with another study (Mohammadian et al., 2019), also exhibited a significant improvement in the liver function following minocycline and ibuprofen administration in BDL. Therefore, as the HE is a secondary complication of cirrhosis, it could suggest that the observed central therapeutic effects of PRP, at least in part, are mediated due to secondary hepatohealing properties.

The beneficial effects of PRP used in this study on the bile duct ligated rats were most likely due to their direct neuroprotective effects on the brain and secondary consequences of liver functional enhancement.

In conclusion, PRP administration ameliorated the signs of memory impairment and improved hippocampal synaptic plasticity induced by the BDL model. According to the findings, these beneficial effects of PRP could be mediated via direct neuroprotective properties as well as indirect hepatohealing effects. Therefore, further experimental research is recommended to be carried out to establish the potential therapeutic effects of PRP in memory impairment, neuronal plasticity defect, and liver function abnormality in HE patients.

## FUNDING INFORMATION

This study was funded by Iran national Science foundation (97013044).

## DATA AVAILABILITY STATMENT

The data that support the findings of this study are available upon request

### PEER REVIEW

The peer review history for this article is available at https://publons.com/publon/10.1002/brb3.2447
